# Characterising RNA secondary structure space using information entropy

**DOI:** 10.1186/1471-2105-14-S2-S22

**Published:** 2013-01-21

**Authors:** Zsuzsanna Sükösd, Bjarne Knudsen, James WJ Anderson, Ádám Novák, Jørgen Kjems, Christian NS Pedersen

**Affiliations:** 1 Bioinformatics Research Center, Aarhus University, Aarhus, Denmark; 2 Department of Molecular Biology and Genetics, Aarhus University, Aarhus, Denmark; 3 Interdisciplinary Nanoscience Center, Aarhus University, Aarhus, Denmark; 4 CLC bio, Finlandsgade 10-12, Aarhus N, DK-8000, Denmark; 5 Department of Statistics, University of Oxford, OX1 3TG, UK; 6 Oxford Centre for Integrative Systems Biology, University of Oxford, OX1 3QU, UK; 7 Department of Computer Science, Aarhus University, Aarhus, Denmark

## Abstract

Comparative methods for RNA secondary structure prediction use evolutionary information from RNA alignments to increase prediction accuracy. The model is often described in terms of stochastic context-free grammars (SCFGs), which generate a probability distribution over secondary structures. It is, however, unclear how this probability distribution changes as a function of the input alignment. As prediction programs typically only return a single secondary structure, better characterisation of the underlying probability space of RNA secondary structures is of great interest. In this work, we show how to efficiently compute the information entropy of the probability distribution over RNA secondary structures produced for RNA alignments by a phylo-SCFG, and implement it for the PPfold model. We also discuss interpretations and applications of this quantity, including how it can clarify reasons for low prediction reliability scores. PPfold and its source code are available from http://birc.au.dk/software/ppfold/.

## Background

The function of RNA molecules is known to depend on their three-dimensional structure, which is stabilized by a secondary structure scaffold of basepairing. The secondary structure is defined by hydrogen bonds between nucleotides, which form across the structure for thermodynamic stability and molecular function. Despite its importance, the accurate prediction of RNA secondary structure remains an unsolved challenge in computational biology.

With the advent of next-generation sequencing technologies and new methods in transcriptomics, an explosively growing amount of biological RNA data is available in public databases such as Rfam [1] and RNA STRAND [2]. This makes it possible to acquire a large number of RNA alignments to be used in comparative RNA secondary structure predictions. This is especially significant in the case of long RNAs such as RNA viral genomes and long genomic introns, many of which are known to have functional, conserved secondary structures.

Several methods have been established to predict RNA secondary structures from nucleotide sequences. In this paper, we focus entirely on non-pseudoknotted secondary structure prediction. Thermodynamic optimisation based on minimising free-energy functions has been used to great effect in algorithms such as mfold [3], UNAFold [4] and RNAfold [5]. In a different approach, stochastic context-free grammars (SCFGs) have also been successfully used to model RNA secondary structure. The Pfold program [6,7], for example, combines molecular evolution with a lightweight SCFG model (known as a *phylo-grammar*) to predict the consensus secondary structure of RNA alignments, and has in the past shown to be highly accurate for structural alignments [8]. PPfold is a recent multithreaded reimplementation of Pfold [9].

Common to these methods is that they produce a probability distribution over all possible nested secondary structures for the input sequences, but usually only a single, optimal secondary structure is reported to the user. A particularly interesting question is how the underlying distribution changes as a function of input data. Due to the large space of possible secondary structures, however, it is difficult to report useful quantities to describe this. Information entropy is one such measure.

Entropy computations in the context of RNA secondary structure prediction have been considered previously from a thermodynamic perspective, calculating the thermodynamic entropy over both secondary structure space [10,11] and tertiary structure space [12]. Positional thermodynamic entropy [5] as well as thermodynamic entropy changes in response to basepair mutations [13] have also been computed. Additionally, SCFG-based methods have recently been utilised for calculating both the information entropy of individual basepairs in a single-sequence context [14], and the thermodynamic entropy changes in response to basepair mutations. However, no form of entropy has been computed previously in the case of comparative RNA secondary structure prediction.

The information entropy *H *of a probability distribution P  with a set of events X  is defined as:

(1) $$H\left(P\right)=-\underset{x\in X}{\sum }P\left(x\right){\text{log}}_{2}\left(P\left(x\right)\right).$$

The information entropy is a measure for the "spread" of the probability distribution, and has well-defined lower and upper bounds. The minimum entropy of 0 occurs when there is only one outcome with probability 1. For *n *possible outcomes, the maximum entropy is equal to log_2 _(*n*) and occurs for the uniform distribution. For a probability distribution, an entropy of *k *bits indicates that the expected value of the information content of observing a single outcome is *k *bits. In the context of secondary structure prediction, a low entropy therefore indicates that few secondary structures dominate the probability space, whereas a high entropy indicates a more even probability distribution over possible secondary structures. Thus, information entropy is a useful single quantity to characterize the underlying probability distribution of secondary structures.

In the case of RNA secondary structure prediction based on a semantically unambiguous SCFG, the information entropy of the probability distribution over RNA secondary structures can be computed as the derivational entropy of the SCFG that generates the distribution. We restrict ourselves to semantically unambiguous SCFGs, in order to maintain a one-to-one correspondence between SCFG derivations and secondary structures. Thus, throughout this paper we use "information entropy" and "derivational entropy" interchangeably.

### Notation

Consider RNA alignments of *k *sequences (*k *≥ 1), with the *i*'th column denoted *c_i _*∈ Σ*^k ^*= {"A", "C", "G", "U", "-"}*^k ^*\ {"-"}*^k^*. A stochastic context-free phylo-grammar (phylo-SCFG) on such alignments is a tuple *G *= ((Σ*^k^, N, S, R*), *P*), where:

• Σ*^k ^*forms the (finite) set of terminal symbols

• *N *is a finite set of nonterminal symbols, such that $\sum^{k}\cap N=0̸$

• *S *is the start symbol, *S *∈ *N*

• *R *is a finite set of production rules, each rule of the form *A *→ *α, A *∈ *N *and *α *∈ (∑*^k ^*∪ *N*)*

• *P *is a function from *R *to real numbers in the interval [0,1]

In the case of a phylo-grammar, *P *can be interpreted as Bayesian probabilities equal to the product of prior probabilities that only depend on the type of rule being used, and a likelihood factor that is typically derived from a phylogenetic model and is a function of the alignment columns. We will return to this more formally later. Furthermore, we assume the grammar is proper, that is ∀*A *∈ *N*: ∑_*π *= (*A *→ *α*) _*P*(*π*) = 1.

Let *d *be a complete (left-most) derivation of the grammar. Informally, a complete derivation is a sequence of production rules, such that starting from the start symbol, and sequentially replacing all nonterminals with a production rule emitting from that nonterminal, a string of terminal symbols is obtained. The probability of *d *is the product of the probability of all rules occurring in *d*:

(2) $$p\left(d\right)=\underset{A\to \alpha }{\prod }P{\left(A\to \alpha \right)}^{f_{d}\left(A\to \alpha \right)}$$

where *f_d _*(*A → α*) is the number of times rule *A → α *occurs in derivation *d*.

The grammar is consistent if ∑*_d _p*(*d*) = 1, where the sum is over all possible derivations of the grammar. In the case of phylo-grammars, consistency implies that the total probability of the grammar emitting all alignments of *k *sequences (of all lengths) is 1.

The expected frequency (count) of a rule *A → α *in all derivations of the grammar is

(3) $$Ef\left(A\to \alpha \right)=\underset{d}{\sum }p\left(d\right)f_{d}\left(A\to \alpha \right).$$

The expected frequency of each rule can be computed in practice using a dynamic programming algorithm known as the *inside-outside algorithm*, as described in [15]. Following the approach of [16], we factorise a complete derivation *d *at each occurrence of rule *A *→ *α *into an "innermost" sub-derivation $d_{2}:\alpha \overset{d_{2}}{\Rightarrow }s$, where *s *∈ (∑*^k^*)*, and two "outermost" sub-derivations $d_{1},d_{3}:S\overset{d}{\Rightarrow }\beta A\gamma ,\beta \overset{d_{1}}{\Rightarrow }t,\gamma \overset{d_{3}}{\Rightarrow }u$, where *β, γ *∈ (∑*^k ^*∪ *N*)* and *t, u *∈ (∑*^k^*)*. Then

(4) Ef(A→α)=O(A)I(α)P(A→α),

with

(5) $$I\left(\alpha \right)=\underset{d}{\sum }\underset{d_{2}}{\sum }p\left(d_{2}\right)$$

(6) $$O\left(A\right)=\underset{d}{\sum }\underset{d_{1},d_{3}}{\sum }p\left(d_{1}\right)p\left(d_{3}\right).$$

The *I*(*α*) and *O*(*A*) variables can also be computed for a particular string *C_l _*of length *l*, using the inside-outside algorithm in *O*(*l*^3^) time.

The derivational entropy of an SCFG is the information entropy of the probability distribution of all derivations under the SCFG (c.f. equation 1):

(7) $$H\left(G\right)=-\underset{d}{\sum }p\left(d\right){\text{log}}_{2}\left(p\left(d\right)\right)$$

where the sum is over all possible derivations of the grammar. This quantity can be computed efficiently using expected rule frequencies [16]:

(8) $$H\left(G\right)=-\underset{A\to \alpha }{\sum }{\text{log}}_{2}\left(P\left(A\to \alpha \right)\right)Ef\left(A\to \alpha \right).$$

We assume that the phylo-SCFG describes RNA secondary structure, and while it may be *syntactically *ambiguous, it is *semantically *unambiguous. In practice, this means that there may be a number of possible derivations for a particular alignment, but there is a one-to-one correspondence between derivations and consensus secondary structures for the RNA alignment. Furthermore, we express RNA structure SCFGs in double emission normal form [17], allowing only rules of the following types:

$$\begin{array}{c} \mathrm{Type}1:A\to c\\ \mathrm{Type}2:A\to cBc^{\prime }\\ \mathrm{Type}3:A\to BC \end{array}$$

for *A, B, C *∈ *N, c, c^' ^*∈ Σ*^k^*. Apart from generating empty strings, all SCFGs modelling nested RNA secondary structures can be written in double emission normal form, so the methods presented here can be adapted to RNA secondary structure grammars of all types. Additionally, they can also be adapted for specific mildly context-sensitive RNA grammars that generate specific types of pseudoknots.

Type 1 rules correspond to the production of a single column of the alignment, and their probability can be expressed as

(9) $$P\left(A\to c\right)=P_{G}\left(A\to c\right)P_{T}\left(c|ċ\right)$$

where *P_G_*(*A *→ *c*) only depends on *A*, and $P_{T}\left(c|ċ\right)$ is the likelihood of observing column *c *under the phylogenetic model, assuming that it is unpaired in the consensus structure (denoted by ċ ).

Type 2 rules correspond to the production of two basepaired columns of the alignment, and their probability can be expressed as

(10) $$P\left(A\to cBc^{\prime }\right)=P_{G}\left(A\to cBc^{\prime }\right)P_{T}\left(c,\;c^{\prime }|\hat{c,c^{\prime }}\right)$$

where *P_G_*(*A *→ *cBc'*) only depends on *A *and *B*, and $P_{T}\left(c,\;c^{\prime }|\hat{c,c^{\prime }}\right)$ is the likelihood of observing column pair *c, c^' ^*under the phylogenetic model, assuming that they are paired with each other in the consensus structure (denoted by $\hat{c,c^{\prime }}$).

Type 3 rules express bifurcation and correspond to dividing the alignment into two parts. As these rules do not depend on alignment columns, we have:

(11) $$P\left(A\to BC\right)=P_{G}\left(A\to BC\right)\cdot 1$$

where *P_G_*(*A *→ *BC*) only depends on *A, B *and *C*.

It is now clear that the probability of any particular derivation under a phylo-SCFG this structure can be expressed as a product of two probabilities: a probability *p_G _*that only depends on the types of rules used, and a probability *p_T _*that only depends on the emitted alignment columns: *p*(*d*) = *p_G_*(*d*) *p_T_*(*d*), with

(12) $$\begin{array}{ccc} p_{G}\left(d\right)= & \underset{r_{a}}{\prod }P_{G}{\left(r_{a}\right)}^{f_{d\left(r_{a}\right)}}\underset{r_{b}}{\prod }P_{G}{\left(r_{b}\right)}^{f_{d\left(r_{b}\right)}} & \\ & \;\;\times \underset{r_{c}}{\prod }P_{G}{\left(r_{c}\right)}^{f_{d}\left(r_{c}\right)} \end{array}$$

(13) $$p_{T}\left(d\right)=\underset{r_{a}}{\prod }P_{T}{\left(c|ċ\right)}^{f_{d\left(r_{a}\right)}}\underset{r_{b}}{\prod }P_{T}{\left(c,\;c^{\prime }|\hat{c,c^{\prime }}\right)}^{f_{d\left(r_{b}\right)}}$$

for *r_a _*∈ *R *of Type 1, *r_b _*∈ *R *of Type 2, *r_c _*∈ *R *of Type 3.

Given a single RNA alignment, there are typically a large number of possible derivations, each corresponding to a possible secondary structure for the alignment. In the rest of this work, we restrict ourselves to this space of derivations, which we characterize by its derivational entropy as described below.

## Results and discussion

### Algorithm

Let the set of all derivations for the input alignment be Φ. The total probability of the grammar producing the input string is:

(14) $$T=\underset{d\in \Phi }{\sum }p\left(d\right)=\underset{d\in \Phi }{\sum }p_{G}\left(d\right)p_{T}\left(d\right).$$

The computation of *T *is straightforward using the inside algorithm. The normalized probability of a derivation *d *is $p_{\Phi }\left(d\right)=\frac{1}{T}p\left(d\right)=\frac{1}{T}p_{G}\left(d\right)p_{T}\left(d\right)$. We now define the information entropy of the input alignment under the phylo-SCFG model as:

(15) $$H_{\Phi }\left(G\right)=-\underset{d\in \Phi }{\sum }p_{\Phi }\left(d\right){\text{log}}_{2}\left(p_{\Phi }\left(d\right)\right).$$

Note that Equations 4 and 8 still hold when the set of derivations is restricted to a subset, as opposed to the entire space, so we can write the entropy as:

(16) $$\begin{array}{ccc} H_{\Phi }\left(G\right) & =\underset{d\in \Phi }{\sum }\frac{p\left(d\right)}{T}{\text{log}}_{2}\left(\frac{T}{p_{G}\left(d\right)p_{T}\left(d\right)}\right) & \\ & =\;{\text{log}}_{2}\left(T\right)-\frac{1}{T}\underset{d\in \Phi }{\sum }p\left(d\right){\text{log}}_{2}\left(p_{G}\left(d\right)\right)\\ & \;-\frac{1}{T}\underset{d\in \Phi }{\sum }p\left(d\right){\text{log}}_{2}\left(p_{T}\left(d\right)\right) \end{array}$$

We now show how to express the entropy in terms of expected rule frequencies. Note that:

(17) $$\begin{array}{c} \underset{d\in \Phi }{\sum }p\left(d\right){\text{log}}_{2}\left(p_{G}\left(d\right)\right)=\\ \;=\underset{A\to \alpha }{\sum }{\text{log}}_{2}\left(P_{G}\left(A\to \alpha \right)\right)Ef\left(A\to \alpha \right) \end{array}$$

which can be computed using the expected rule frequencies obtained from the inside-outside algorithm (cf. equations 7 and 8). This can be seen by noting that Equations 4 and 7 still hold when the set of derivations is restricted to a subset, as opposed to the entire space.

Furthermore, if $1^{d}\left(i,\;j\right)$ denotes the indicator function for whether the column pair (*i, j*) is emitted from a Type 2 rule (i.e. position *i *and *j *form a pair), and $1^{s}\left(i\right)$ denotes the indicator function for whether column *i *is emitted from a Type 1 rule (i.e. position *i *is unpaired), then:

(18) $$\begin{array}{c} \underset{d\in \Phi }{\sum }p\left(d\right){\text{log}}_{2}\left(p_{T}\left(d\right)\right)=\\ \;=\;\underset{d\in \Phi }{\sum }p\left(d\right)\left(\underset{r_{a}}{\sum }{\text{log}}_{2}\left(P_{T}{\left(c|ċ\right)}^{f_{d}\left(r_{a}\right)}\right)\right)\\ \;\;\;+\left(\underset{r_{b}}{\sum }{\text{log}}_{2}\left(P_{T}{\left(c,\;c^{\prime }|\hat{c,c^{\prime }}\right)}^{f_{d}\left(r_{b}\right)}\right)\right)\\ \;=\underset{\begin{array}{c} i,j\\ i\neq j \end{array}}{\sum }{\text{log}}_{2}\left(P_{T}\left(i,j|\hat{i,j}\right)\right)\underset{d\in \Phi }{\sum }1^{d}\left(i,\;j\right)p\left(d\right)\\ \;\;\;+\underset{i}{\sum }{\text{log}}_{2}\left(P_{T}\left(i,|\dot{i}\right)\right)\underset{d\in \Phi }{\sum }1^{s}\left(i\right)p\left(d\right) \end{array}$$

We observe that $\sum_{d\in \Phi }1^{d}\left(i,\;j\right)p\left(d\right)$ is just the total probability under the model that positions *i *and *j *form a pair (i.e. they are emitted from rule type 2), and $\sum_{d\in \Phi }1^{s}\left(i\right)p\left(d\right)$ is just the total probability under the model that position *i *is unpaired (i.e. it is emitted from rule type 1). The quantity ∑_*d*∈Φ _*p*(*d*) log_2_(*p_T_*(*d*)) can therefore also be computed using the expected rule frequencies.

Once the values of the inside-outside variables have been calculated for an input string of length *n*, the expected rule frequencies can be computed in *O*(*n*) time for rules of Type 1, *O*(*n*^2^) time for rules of Type 2, and *O*(*n*^3^) time for rules of Type 3. As the time complexity of the inside-outside algorithm is also *O*(*n*^3^), the computation of the entropy over the possible derivations of the input string does not increase the time complexity of RNA secondary structure prediction.

### Interpretation of the derivational entropy

The derivational entropy provides a measure for the "spread" of the probability distribution on possible secondary structures. For equiprobable events, information entropy increases logarithmically with the number of possible outcomes. It is clear, therefore, that the maximum derivational entropy increases with sequence length. It has been shown [18] that, assuming all nucleotide pairings are possible, the number of secondary structures *S*(*l*) of length *l *can be approximated for large *l *as:

(19) $$S_{l}\approx 1.104\times l^{-\;\frac{3}{2}}\times 2.618^{l}.$$

The maximum derivational entropy is therefore expected to increase logarithmically with *S_l_*.

(20) $$H_{\max}\left(l\right)\approx 0.142-\frac{3}{2}{\text{log}}_{2}\left(l\right)+1.388l.$$

*H_max_*(*l*) provides an upper bound on the value of the information entropy for an RNA alignment of length *l*, and can aid the user in the interpretation of the entropy corresponding to a particular input alignment.

**Figure 1:**
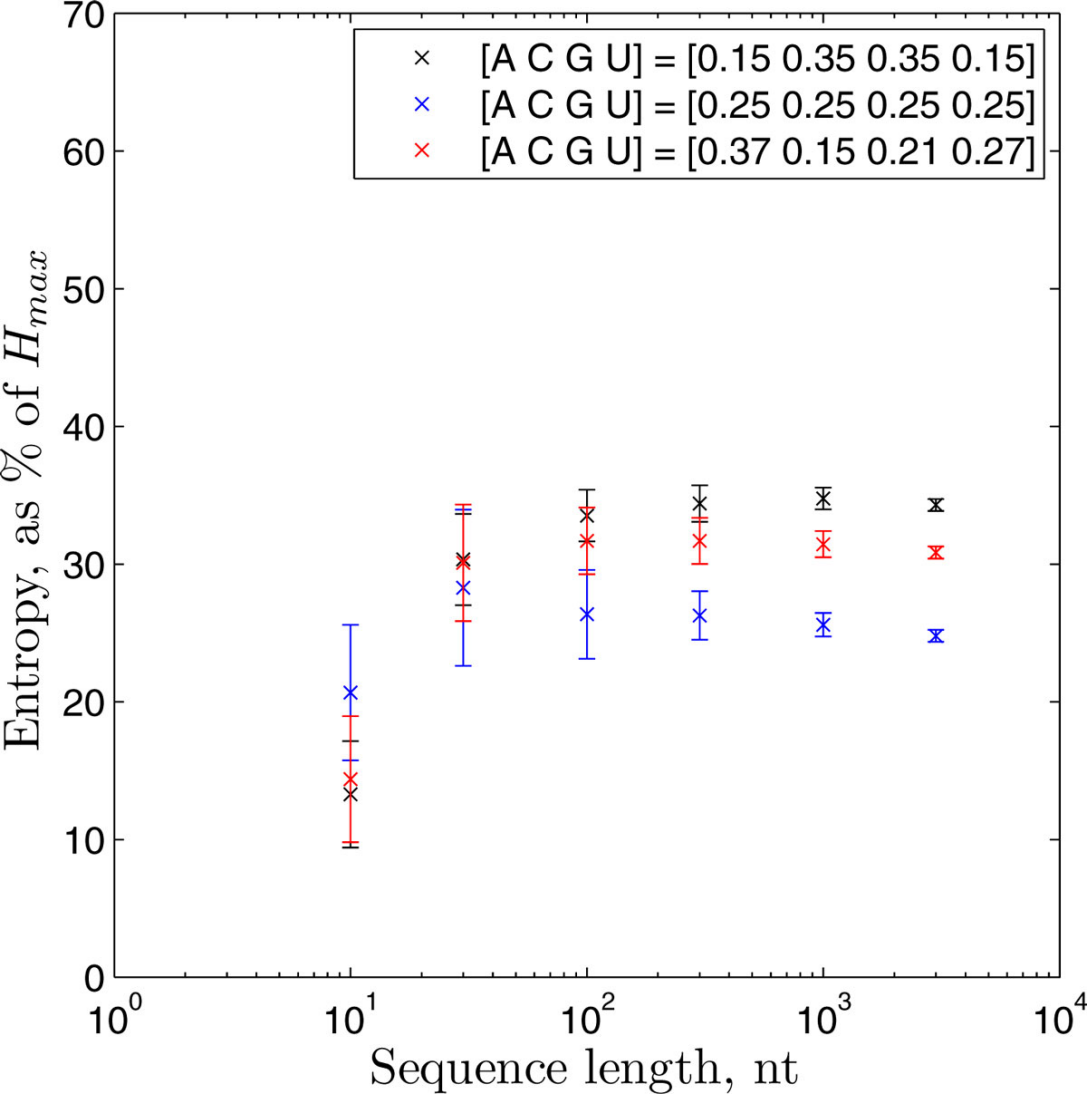
**The entropy of the structure probability distributions computed by PPfold, for random sequences of various lengths and nucleotide compositions**. The nucleotide composition of each dataset is given in the legend. Each point represents the mean of the entropy values for 100 random sequences, with the error bars indicating the standard deviations.

In practice, however, *H_max _*is rarely attained by nucleotide sequences. To obtain more intuition for the value of the derivational entropy, we generated random nucleotide sequences of varying lengths and nucleotide compositions, and computed the entropy of the probability distribution generated by PPfold for the single-sequence predictions, as a percentage of the theoretical *H_max_*(*l*) (Figure 1). Interestingly, we found that the entropies computed for these random sequences are remarkably stable at around 25-35% of *H_max_*(*l*) over a wide range of sequence lengths (> 30 nucleotides), with only a slight dependence on nucleotide composition. Particular entropy values can therefore be interpreted in relation to this; an entropy value of 25-35% of *H_max_*(*l*) suggests that the probability distribution for the input data is as "spread" over the structure space as it would be for a single random sequence of that length.

### Using information entropy to interpret low reliability scores

Derivational entropy is related to various reliability measures already reported by prediction programs. PPfold in particular computes the probability of a pair between two columns as the sum of the (normalized) expected frequencies of rules of Type 2 emitting that column pair:

(21) $$P\left(\hat{i,j}\right)=\underset{N1\to cN_{2}c^{\prime }}{\sum }O\left(N_{1}\right)I\left(N_{2}\right)P_{G}\left(N_{1}\to cN_{2}c^{\prime }\right)P_{T}\left(i,j|\hat{i,j}\right).$$

The probability of column *i *remaining unpaired is $P\left(\dot{i}\right)=1-\sum_{\begin{array}{c} i,j\\ i\neq j \end{array}}P\left(\hat{i,j}\right)$. These probabilities function as "reliability scores" for every predicted base-pair or unpaired nucleotide in the structure. The overall reliability score for the secondary structure can be computed as the average of the reliability scores of all positions.

Importantly, while the reliability scores depend both on the structures and their probabilities, the derivational entropy is only a function of probabilities, and does not depend on the similarity of the structures to each other. Derivational entropy therefore provides complementary information to reliability scores. For example, if the reliability scores predict a low accuracy, the entropy can help reveal the underlying reasons. A low reliability score can be observed in different situations, for example (a) if there is insufficient structure signal, so there are no structures of high probabilities and the probability distribution is "spread", or (b) if there are two or more possible (topologically different) structures of high probabilities, so the probability distribution has several "peaks". Entropy will be high in the first case, but low in the second case, and can therefore be used to distinguish the two situations from each other.

To illustrate this with a practical example, a PP-fold prediction of the secondary structure of the random nucleotide sequence:

GACCAAACGCAGCCAGCGTCACTGTAGGATTTAAA

ACCGAGGGAATGCCGTCAGTAGGGTCGGGTTTAAC

reveals that the underlying probability distribution has an entropy of 28.55 bits, with an average reliability score 0.65 for the final predicted structure. In comparison, the following combinatorial sequence of the same length:

GGGGAAACCCCAAAGGGGAAACCCCAAAGGGGAAA

CCCCAAAGGGGAAACCCCAAAGGGGAAACCCCAAA

shows a significantly lower entropy of 18.82 bits, at the same time as a low average reliability score of 0.56. Despite the low reliability scores in both cases, the entropy value correctly reveals that the combinatorial sequence has a relatively small number of very different secondary structures dominating the probability space, whereas the probabilities are more uniformly distributed over a large number of possible structures in the case of the random sequence.

**Figure 2:**
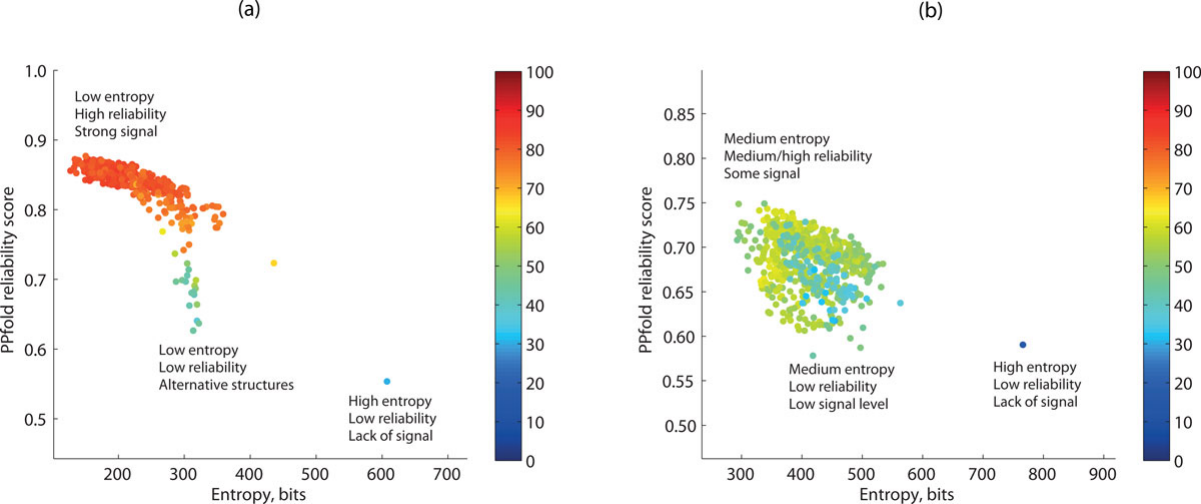
**Use of entropy in conjunction with reliability scores to characterise probability distributions on RNA structure space**. (a) Prokaryotic alignments (*H_max _*≈ 2102), (b) Eukaryotic alignments (*H_max _*≈ 2471). The color scale reflects the accuracy of each prediction (F-measure, %). The outlier with the largest entropy value in each case corresponds to the structure prediction on a single sequence.

To investigate if similar patterns could also be observed for alignments of longer biological sequences, we applied the same technique on a range of bacterial and eukaryotic 16S/18S rRNA alignments. The generation of the alignments is described in the Methods section. We plotted the entropy against the PPfold reliability scores, and colour-coded the data points after the accuracy of predictions. As Figure 2 shows, entropy can be used in conjunction with basepairing probabilities to evaluate RNA secondary structure prediction results even in the case of long biological sequences. In the cases where reliabilities are high and entropy is low, the probability space is dominated by a clearly defined set of similar structures, which are likely to be well predicted. In the cases where basepairing probabilities are low, a simultaneous high entropy implies that the underlying probability distribution lacks a clear signal (eg. in the case of folding just one sequence). By contrast, low reliabilities coupled to a low entropy suggest a distribution that has several competing structures dominating the probability space.

### Relationship to prediction accuracy

An important question with respect to comparative RNA secondary structure prediction is how the accuracy of predictions varies with the quality of input alignment, and to what extent the accuracy of a predicted structure can be predicted. Reliability scores and entropy both measure variation in the secondary structure space, so both are expected to be correlated with prediction accuracy. We note, however, that a natural limitation of both entropy and reliability scores is that they are computed under the model, which effectively assumes that the model itself is an accurate description of the biological folding processes. If this is not the case, high confidence values computed under the model can still correspond to low prediction accuracies in reality.

**Figure 3:**
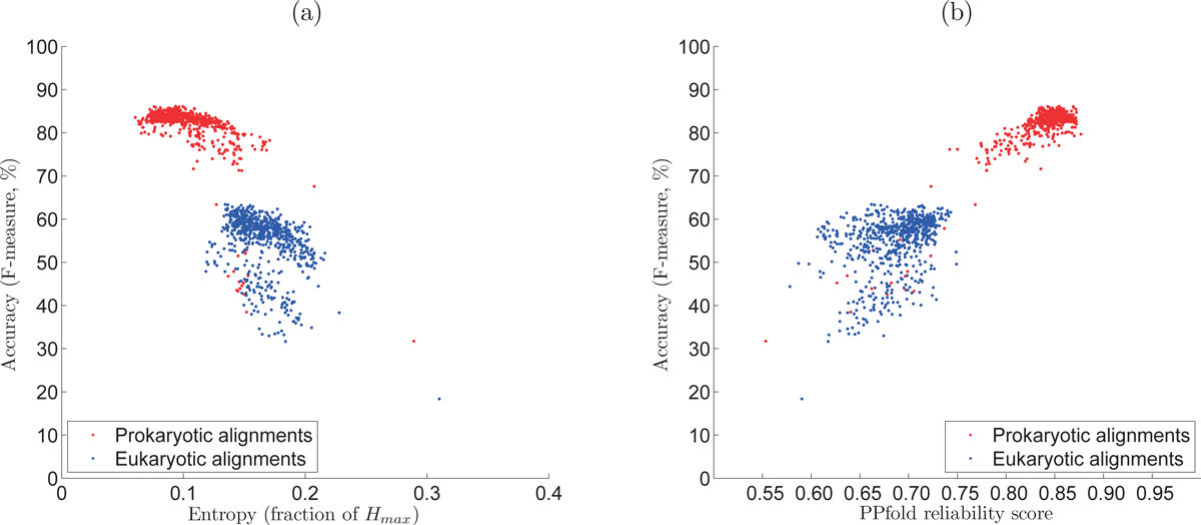
**Variation in accuracy (F-measure) correlated with (a) derivational entropy (as fraction of *H_max_*) and (b) PPfold reliability scores, for prokaryotic and eukaryotic 16S/18S rRNA alignments**.

Nevertheless, we compared how the accuracy of predictions correlates with both the average structure reliability scores and the information entropy, for all alignments in our dataset consisting of bacterial and eukaryotic 16S/18S rRNA alignments. The results are shown in Figure 3. As expected, both the PPfold reliability scores and entropy are correlated with prediction accuracy, for both bacterial and eukaryotic alignments. The correlations are stronger in the case of the bacterial alignments and weaker in the case of the eukaryotic alignments. In the case of the eukaryotic alignments, entropy appears to be slightly better correlated with prediction accuracy than PPfold reliability scores, although the difference is not statistically significant.

We observe that despite the PPfold reliability scores generally suggest somewhat higher prediction accuracies than what was actually observed, they convey *absolute *information about the accuracy of predictions. Instead, entropy functions as a *relative *accuracy measure, when comparing several structure predictions for the same sequence. Entropy can, therefore, complement the currently existing measures for an increased understanding of RNA structure variability.

Lastly, we note that there is large variation in the reliability measures reported by different RNA structure prediction programs. By contrast, information entropy is a *method-independent *measure: for the same alignment, it will have the same, well-defined interpretation, regardless of the method producing the underlying probability distributions. Additionally, all classic information theoretic results can be applied to it. Information entropy can therefore be used to compare the behaviour of any secondary structure prediction methods that produce a probability distribution over RNA secondary structures, in a way that is not possible with currently existing methods.

Future perspectives include the computation of measures similar to entropy, such as the self-information of particular outcomes, or the Kullback-Leibner divergence of distributions to compare constrained and unconstrained models for RNA folding. We also expect that entropy may provide a number of other possible future applications in RNA secondary structure prediction.

## Conclusions

The information entropy of the probability distribution generated by phylo-grammars can be computed efficiently from the inside-outside variables, and has been implemented as part of PPfold. Information entropy is a well-defined characteristic of the underlying probability distribution, which complements the reliability values already reported by algorithms for an increased understanding of RNA structure variability. It is also a method-independent measure of prediction certainty, providing theoretical advantages over existing methods.

## Methods

### Implementation

We have implemented the algorithm for the Knudsen-Hein (KH99) grammar [6] in PPfold [9]. PPfold has been written in Java 6.0 and is available as a standalone application. The rules of the KH99 grammar are as follows:

$$\begin{array}{ccccc} S & \to & L & | & LS\\ L & \to & c & | & cFc^{\prime }\\ F & \to & cFc^{\prime } & | & LS \end{array}$$

We note that the KH99 grammar is not in double emission normal form, as it includes the rule *S → L*. Even though the grammar can be expressed in the desired form, adapting the algorithm to include this additional rule is both straightforward and highly efficient; this is therefore what we have implemented.

In the case of the KH99 grammar, there is a maximum of one bifurcating rule originating from each nonterminal symbol. It is also known that the expected frequency of a nonterminal symbol can be computed in *O*(*n*^2^) time for any SCFG, and the expected frequencies of rules from the same nonterminal symbol sum to the expected frequency of the nonterminal symbol. Hence, in the case of the KH99 grammar, the time complexity of the computation of the derivational entropy (given the inside-outside variables) could further be reduced to *O*(*n*^2^).

As described in the Results and Discussion section, the value of the entropy depends on the length of the alignments. Hence, small adaptations were made in the algorithm to be able to compare the entropies of alignments that include a particular sequence. The default option in PPfold is to remove columns where fewer than 75% of the sequences have nucleotides. In the case of entropy computation, this is replaced with removing only the columns where the selected sequence has gaps. This ensures that all alignments that include the selected sequence have an equal prediction length.

### Test sequences and alignments

Random sequences of different nucleotide compositions were generated using the online FaBox tool [19].

For the generation of alignments, among families with an experimentally verified secondary structure in the Rfam database, we chose two families (RF01960 - Eukaryotic small subunit ribosomal RNA, and RF00177 - Bacterial small subunit ribosomal RNA), for which our initial tests indicated that a particularly wide range of structure prediction accuracies may be achieved depending on the choice on the sequences included in the alignment. Starting with a sequence of interest in both families, we constructed our datasets by randomly adding sequences from the family alignment one by one, up to a final size of maximum 15 sequences. For each alignment size (between 1 and 15 sequences in the alignment), the process was repeated 50 times. This way we obtained 1+14 × 50, not necessarily distinct cases per family, with 1-15 sequences each (including the starting sequence as a standalone case). The alignments of the selected sequences and the reference secondary structures thereof were adapted from those in Rfam, by deleting gap-only columns and any base pairs involved with those columns.

### Comparing accuracies

Accuracies are reported in terms of the *F-measure*, which is the harmonic mean of the sensitivity and the positive predictive value (PPV) of the basepair (bp.) predictions, compared to the comparative (reference) structure. These quantities are defined as:

(22) $$\text{sensitivity}=\frac{TP}{\text{number of bp}\text{. in reference}}$$

(23) $$\text{PPV}=\frac{TP}{\text{number of bp}\text{. in prediction}}$$

(24) $$\text{F}-\text{measure}=2\times \frac{\text{sensitivity}\times \text{PPV}}{\text{sensitivity}+\text{PPV}}$$

where *TP *is the number of correctly predicted base-pairs.

## Competing interests

The authors declare that they have no competing interests.

## Authors' contributions

ZS designed the algorithm with the help of BK and CNSP, implemented it and wrote the manuscript. JWJA and ÁN provided testing data, participated in the testing, and helped with writing the manuscript. CNSP and JK supervised the project, critically revised the manuscript and provided funding. All authors read and approved the final manuscript.

## Declarations

This research has been funded by the Danish Council for Strategic Research under the project #09-061856. JWJA would like to thank the EPSRC for funding. ÁN would like to thank BBSRC for continued funding through OCISB. CNSP is supported by Centre for Membrane Pumps in Cells and Disease (PUMPKIN), a Centre of Excellence funded by the Danish National Research Foundation. The publication costs for this article were also funded by PUMPKIN.

This article has been published as part of *BMC Bioinformatics *Volume 14 Supplement 2, 2013: Selected articles from the Eleventh Asia Pacific Bioinformatics Conference (APBC 2013): Bioinformatics. The full contents of the supplement are available online at http://www.biomedcentral.com/bmcbioinformatics/supplements/14/S2.
